# Narrative Review of Web-based Healthy Lifestyle Interventions for Cancer Survivors

**DOI:** 10.19080/arr.2020.05.555670

**Published:** 2020-03-10

**Authors:** Victoria Williams, Nashira Brown, Alahni Becks, Dori Pekmezi, Wendy Demark-Wahnefried

**Affiliations:** 1 Department of Health Behavior, University of Alabama at Birmingham (UAB), Birmingham, Alabama, USA; 2 Tuskegee University, Tuskegee, Alabama, USA; 3 Department of Nutritional Sciences, UAB, Birmingham, Alabama, USA; 4 UAB Comprehensive Cancer Center, Birmingham, Alabama, USA

**Keywords:** Cancer survivors, Web-based, Lifestyle intervention, Nutrition, Physical activity

## Abstract

This narrative review of web-delivered weight management, diet quality, and physical activity interventions for cancer survivors relies on a systematic search of PubMed, Psych Info, and EBSCOhost which identified 19 unique web-delivered lifestyle interventions for cancer survivors. The sample sizes for these studies ranged from 11–492. Intervention duration ranged from 1–12 months; however, most interventions were 6–12 weeks in length. Ten studies were randomized controlled trials (RCTs), two were two-arm quasi RCTs, and seven employed a single-arm pre/post-test design. Many (N= 15) of the interventions were well-grounded in behavioral theory, which may have led to favorable behavior change. Most studies (15-of-19) targeted and reported increases in physical activity, while only a few targeted and reported improvements in diet quality (36.9% and 15.8%, respectively) and weight management (26.3% and 10.5%, respectively). A notable limitation was that most studies were conducted among populations that were primarily White and female. Future directions for Internet-based lifestyle interventions for cancer survivors include increasing: (a) focus on multiple behavior change, (b) representation of male and minority populations to improve generalizability of findings, (c) extended intervention duration and follow-up to evaluate long-term efficacy of web-based lifestyle interventions, and (d) sample size to allow for adequate statistical power.

## Introduction

By the year 2029, there will be an estimated 21.7 million cancer survivors [1]; a population increasingly growing due to the aging population, improved early detection, and modern therapeutics and treatment modalities [1]. However, cancer survivors represent a vulnerable population characterized by high rates of obesity, physical inactivity, poor diets, and related chronic conditions [2–4]. Face-to-face lifestyle interventions have shown promise for improving the quality-of-life and reducing adverse health outcomes among cancer survivors [5–8]. However, these approaches are relatively costly and thus have limited reach to the survivors who need them most [8]. Web-delivered lifestyle interventions have already shown success in other populations [9,10] and have the potential to reach large numbers of cancer survivors at relatively low cost. Moreover, web-based strategies can overcome obstacles cited by cancer survivors by not requiring transportation or clinic visits [8]. In fact, they can be accessed 24/7 from any Internet-enabled device [11–13]. Past studies indicate that cancer survivors are already using the Internet to obtain healthy lifestyle information [14] and report a lack of credible online resources for healthy lifestyle behavior change information [15]. Thus, there is an evident interest and need for web-based lifestyle intervention in this patient population.

Findings-to-date from studies in this area have been encouraging. Prior related reviews on non-face-to-face lifestyle interventions (including only three web-based interventions) [16], eHealth physical activity interventions [17], and digital diet and physical activity interventions for cancer survivors [18] all found the number of health behavior change interventions are increasing. It is important to note that digital and eHealth interventions are distinct from web-based interventions. Web-based interventions require an Internet connection for program delivery, whereas eHealth interventions use the Internet specifically to provide healthcare services, while digital programs do not require the Internet for intervention delivery (e.g., Gaming systems like Wii can be operated by inserting a game into a console without an Internet connection). Nevertheless, there has yet to be a review of web-based physical activity and diet interventions (as both behaviors are critical to maintaining a healthy weight) for cancer survivors. Therefore, there is a need for a review of web-based lifestyle intervention research in cancer survivors to help identify the next steps and move this field forward, given the critical public health implications and rapidly evolving nature of the Internet.

## Methods

### Study Design

The guidelines of the “Preferred Reporting Items for Systematic Reviews and Meta-Analyses” (PRISMA) [19] were followed to systematically search the literature to identify studies examining web-delivered lifestyle interventions for cancer survivors. The search was conducted for papers published through August 10, 2019.

### Study Selection

PubMed, Psych Info, and EBSCOhost were systematically searched for articles published on web-based nutrition, physical activity, and/or weight management interventions for adult cancer survivors. The research intersected terms for physical activity (exercise), weight (weight loss, weight management, weight change, weight reduction), nutrition (diet), Internet (web, web-based), cancer (cancer survivorship, tumor, neoplasm) and interventions (programs). Duplicate papers, papers not written in English, those containing non-human and non-adult samples were manually removed through the study screening process. Included studies met the following criteria: web- or Internet-based programs requiring an Internet connection to access intervention materials (e.g., sending or receiving an email, visiting a website or accessing a mobile application) and targeting weight management, physical activity, and/or nutrition in adult cancer survivors diagnosed with any cancer type during or post-treatment.

Studies were excluded for the following reasons: a) study was not exclusively for cancer survivors; b) non-web or Internet-based studies; c) not in English; d) only available as an abstract; d) overlapping publications; e) protocol papers, and f) review papers. Intervention studies with quasi-experimental and pre-/ post-test research designs were included as this is an emerging field. Those early studies may help shed light on future directions and provide a holistic view of current research. Two researchers independently identified and reviewed studies potentially meeting eligibility criteria. First, the titles and abstracts were reviewed, and duplicate articles were removed. For studies passing the initial screening, the article’s full text was reviewed. A data extraction form was used to record pertinent information on each study consistently. The form included sections on the papers’ authors, country of origin, publication year, study design, cancer type, intervention activities, number of participants, intervention duration, and behavioral outcomes. Any discrepancies were resolved by consensus or through discussion with a third researcher.

## Results

**Figure 1.**
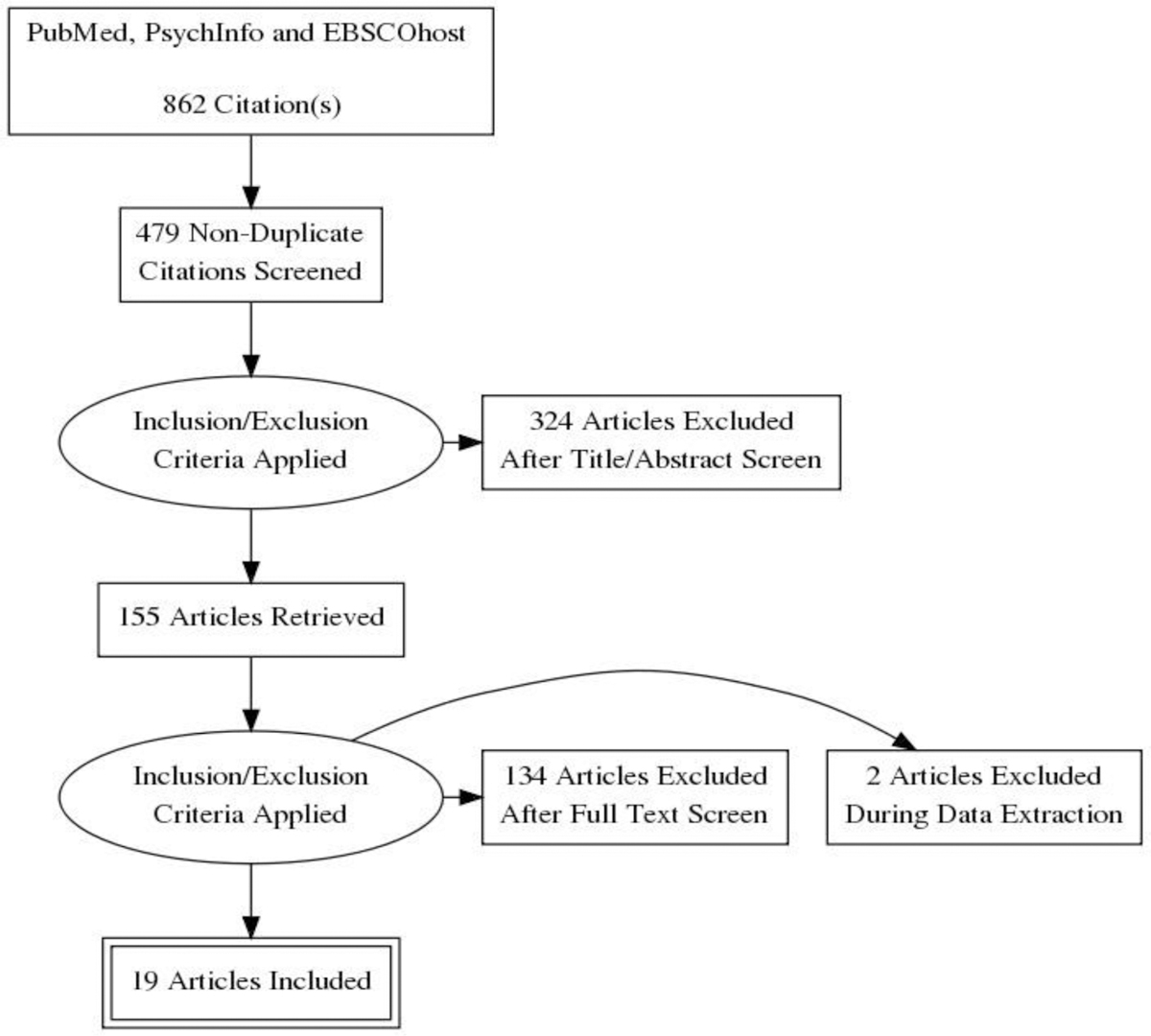
PRISMA flow diagram illustrating article selection strategy

See Figure 1 for the PRISMA flow diagram of the study selection process. The search strategy initially identified 862 records; 19 unique web-based lifestyle interventions for cancer survivors met the inclusion criteria and were included in this review. The included articles were published between 2011 and 2018. Table 1 provides a summary of each of the studies included in the review.

### Participant characteristics:

The number of study participants ranged from 11 to 492 cancer survivors. Fourteen studies [20–33] had a sample size less than 100, and six studies [24,25,27,30–32] enrolled 49 participants or less. Participants mean age ranged from 23 to 73.2 years of age. Three interventions were conducted specifically among young adult cancer survivors [20,25,31], one of which was for young adult cancer survivors diagnosed in childhood [31]. Another study was explicitly for older adult cancer survivors [27]. Six countries were represented in the review. Most studies (N=9) were conducted in the United States [20,21,25–27,29–31,34], followed by the Netherlands (N=3) [33,35,36], Australia (N=2) [23,37], and South Korea (N=2) [28,38]. Other countries represented in the review include Canada [22,24], and the United Kingdom [32].

Most studies (N=12) included both male and female cancer survivors [20,22,23,25,27,30–36]. However, breast cancer survivors were over-represented in these samples. Six studies were exclusively for breast cancer survivors (100% female) [21,26,28,33,37,38], one study was for breast and endometrial cancer survivors (100% female) [29], two for breast, colorectal, and prostate cancer survivors (56% and 82% female) [22,32], and another with testicular and breast cancer survivors (65% female) [30]. Additionally, prostate cancer survivors were also targeted as there was one study exclusively for prostate cancer survivors (100% male) [24], and another with colorectal and prostate cancer survivors (87% male) [36]. As for minority representation, most participants in these studies were White. Only one study included a substantial number of racial and ethnic minorities (83% African Americans, 11% Hispanics, and 6% mixed population) [26]. Other studies (N=6) did not describe the ethnic/racial makeup of their population [28,33,35–38].

### Study design, duration and attrition:

Of the 19 studies, 10 were randomized controlled trials (RCTs) with two [20–22,25,28, 34–36] or three arms [37]. Two more were two-arm quasi RCTs [23,38] due to lack of blinding [23] and randomization practices (participants were assigned based on whether they owned a smartphone) [38]. Seven studies had a single arm pre/post-test design [24,27,29–33].

The RCTs had a variety of control conditions. In the two-arm RCTs, the control groups were either wait-listed [21,23,34,35], received self-help materials related to the intervention (e.g., physical activity information) [20,28], usual care [22,36,38], or health information not directly related to the intervention [25]. The three-arm RCT assessed the relative efficacy of three different website delivery schedules (three intervention modules monthly vs. three intervention modules weekly vs. one intervention module) [37]. Intervention duration ranged from 1–12 months, but most programs (N=15) were short-term (6–12 weeks) [20–29,34]. Only four studies [23,24,34,36] had follow-up post-intervention. Two studies intervened for 12-weeks and assessed outcomes at 12 weeks as well as three months post-intervention [23,24]. Other studies followed-up six months after intervening for six weeks [34] and followed up two months after a 16-week intervention [36]. The overall percentage of dropouts ranged between 0% to 68% with most (n=16) studies reporting attrition rates ≤ 30%.

### Intervention targets and components:

All of the included web-based lifestyle studies intervened on physical activity as either a primary [20–23,25–28,31–38] or secondary objective [24,29,30]. Studies also targeted diet (N=7) [26,28–31,34,35] and weight management (N=5) [23,29,30,32,38], as well as smoking [31,35], depression [34], fatigue [34], and quality of life [38]. Across the studies, the Internet was used in a variety of ways to deliver healthy lifestyle interventions to cancer survivors. Intervention modalities included program websites (N=12) [20,22,23,25,26,28–31,33–37], mobile applications [24,27,29,32,38], email [21,22,25,26,31], and Facebook [20]. Some studies (N=7) used more than one Internet-based method [20,22,25,26,29–31] by combining a website with social media (i.e., Facebook) [20], a mobile application [29], or email [22,25,26,30,31].

Only one intervention relied solely on email for intervention delivery [21]. Intervention emails included healthy lifestyle tips [21,26], feedback based on emailed responses to open-ended questions [21], healthy lifestyle goal recommendations [26], healthy lifestyle educational modules [31] and healthy lifestyle behavior reminders [26,30] (e.g., logging weight [26,30] and physical activity [26]). Common website features/components included goal setting [20,22,23,25,28,29,33–37], self-monitoring (physical activity, weight, or diet) [20,22,25,28,29,33,34,37], and tailored feedback [20,28,29,31,33,35–37]. Websites also provided educational healthy lifestyle modules [22,31,37] (e.g., benefits of exercise, exercise safety, relapse prevention, and building a support network). Mobile applications had similar features as intervention websites as they offered goal setting [27,29,32], self-monitoring(physical activity, weight, or diet) [24,27,29], social networking (e.g., discussion forum where cancer survivors can communicate with other survivors) [27], physical activity plans [32], and tailored feedback [27]. Three studies used commercially available healthy lifestyle websites and applications (e.g., LoseIt [29], Lean Eating [30], and GAIN Fitness [32]), which were not explicitly designed for cancer survivors [29,30,32].

### Measuring behavior change:

Diet quality was assessed using the Dutch Standard Questionnaire on Food Consumption [35], a study generated questionnaire with 35 commonly consumed foods identified as significant contributors to the intake of added sugars, fruits and vegetables, and saturated and trans fats in the National Health and Nutrition Examination Survey [26], the Block Food Frequency Questionnaire [34], three- day dietary recall that assessed diet with the Diet Quality Index (DQI) [28], and the Loselt application [29]. Studies also relied on self-report to measure changes in physical activity except for three studies where accelerometers (e.g., Acti Graph [24,36]) and pedometers [24] (e.g., New Lifestyles [23]) Jawbone [24]) were used. Selfreport changes in physical activity were measured with the Godin Leisure-Time Exercise Questionnaire (GLTEQ) [20,22,32,34,37], the Seven-Day Physical Activity Recall (PAR) [21,25,30], the Short Questionnaire to Assess Health-Enhancing (SQUASH) Physical Activity [35,36], the LoseIt application [29], and the International Physical Activity Questionnaires (IPAQ – both the original and short forms) [33,38], and study generated questionnaires [26–28,31]. Of the study created physical activity instruments, only two were adapted from existing measures (e.g., Behavioral Risk Factors Surveillance System [31] and Cross-Cultural Activity Participation Study [CAPS] [26]). Participants were weighed using scales [23,29,30,32,38].

### Behavior change outcomes:

All included studies targeted physical activity and most (N = 15) reported increases in physical activity [20–22,24–29,32,34–38]. Increases in physical activity ranged from 31 to 126 minutes/week (for studies that reported mins/week) [20,22–26,32,34–37]. Other studies indicated an increase in metabolic equivalents (METs) [38] or the frequency of participants being physically active [21,27,28] (e.g., number of people engaging in ≥ 150 minutes physical activity per week or the number of days of physical activity). Of the studies that reported an increase in physical activity, one study found that both the intervention and self-help comparison group increased self-reported physical activity [20]. Five studies examined weight management among adult cancer survivors [23,29,30,32,38] with three reporting no weight loss [23,32,38], one reporting statistically significant weight improvements at six and 12 months [30], and another finding clinically significant weight loss at four weeks [29]. Of seven studies targeting diet composition [26,28–31,34,35], three found improvements in vegetable and fruit intake [26,28,35], fiber [26], saturated fat [26], and trans-fat [26]. The other four studies found no change in diet [29–31,34].

### Theoretical framework:

The majority of the studies (15-out-of-19) reported using theoretical framework(s) to guide their intervention, which included: Social Cognitive Theory (SCT) [20,21,23,25,26,30,33,37,39], the Transtheoretical Model (TTM) [25,26,28,36], the Theory of Planned Behavior (TPB) [22,35], the Theory of Reasoned Action (TRA) [31], Goal Setting Theory [26,27,36], Social Marketing Theory [26], Self-regulation Theory [35], the I-Change Model [35,36], the Health Belief Model (HBM) [36], the Precaution Adoption Process Model (PAPM) [36], the Health Action Process Approach [36], and Theories of Self-regulation [36]. Eleven studies [20–22,27,28,30,31,33,37,39] used one theoretical framework, while others (N=4) [25,26,35,36] used multiple theories/models. SCT was the most commonly cited model in this review (N=10) [20,21,23,25,26,30,33,37,39], and TTM was used in four studies [25,26,28,36]. Most studies (N=10) [20,21,25,26,28,30,33,35–37] provided detailed description of how theory was incorporated in intervention development.

## Discussion

Internet-based approaches to promote behavioral change in cancer survivors appear promising. All the included studies targeted physical activity, and most were associated with increases in physical activity among cancer survivors. Few interventions targeted weight management and diet quality. However, an estimated 20% of cancer cases and 30% of cancer deaths are attributed to the combined effects of an unhealthy diet, excess body weight, and physical inactivity [40,41]. Lifestyle behaviors are modifiable cancer risk factors that can be addressed to improve the quality and quantity of life among cancer survivors [42]. Therefore, future interventions should target a combination of physical activity, weight management, and diet quality as cancer survivors often require multiple behavior changes to improve their quality of life [43,44]. Behavior change theory has been shown to improve the effectiveness of health behavior change interventions [45–47]. Yet, the descriptions of the theoretical framework used for intervention development varied in detail. Most studies explained how the theory was used for program development (e.g., theoretical constructs were targeted by specific intervention strategies and incorporated into the assessment), but some did not. Future web-delivered lifestyle interventions for cancer survivors should continue using theoretical frameworks for program development and explicitly report how theory is integrated.

Studies included in this review generally focused on short-term improvements in health behavior and tended to forgo following up post-intervention to see if these changes were maintained. While short-term change is favorable, long-term health behavior modifications have lasting health implications (e.g., reduced cancer and recurrence risk). Maintaining healthy lifestyle behaviors is often a challenge as people return to unhealthy lifestyle behaviors [48]. Nevertheless, lengthier interventions and follow-up periods are needed to determine whether web-delivered lifestyle interventions facilitate long-term behavior change. Small sample sizes also were a major limitation of many of the studies that were reviewed. Among the RCTs, many did not appear to have appropriate sample sizes for statistical power. Quasi-RCTs and single-arm pre-/post-test study designs were included in this review as they highlight ongoing research in the field. However, these studies were also typically underpowered. Therefore, future research should include larger sample sizes for greater statistical power to detect intervention effects, especially among segments of cancer survivors.

Lifestyle interventions for cancer survivors have been predominately for cancer-specific populations [16]. A prior review of broad-reach (comprised of telephone, print, and web intervention) lifestyle interventions for cancer survivors found that most programs were conducted with single cancer populations (i.e., exclusively for breast or prostate cancer survivors [16]. However, unhealthy lifestyle behaviors are present and problematic across all cancer types and require appropriate interventions [1]. Encouragingly, this review found that web-delivered lifestyle intervention studies included more “diagnosis diverse” (cancer survivors of more than cancer-type) populations than tend to be reported for face-to-face clinic-based interventions. Of the 19 studies included in the review, 12 studies included diagnosis diverse populations with three studies targeting two populations of cancer survivors (e.g., breast and endometrial; colorectal and prostate; and testicular and breast) and two studies targeting three populations of cancer survivors (e.g., breast, colorectal, and prostate). Therefore, only seven studies targeted single diagnosis populations (e.g., breast and prostate cancer survivors). Despite cancer diagnosis diversity among participants, populations were still predominately female. For example, Puszkiewicz et al. included breast, prostate, and colorectal cancer survivors and the population was 82% female. Therefore, future studies should target diagnosis diverse male cancer survivors. Studies with diagnosis diverse populations and male representation increase the generalizability of findings among cancer survivor groups, as does minority representation.

There is still a lack of participation from ethnic and racial minorities in healthy lifestyle cancer research. Several studies (N=6) did not report the racial/ethnic distribution of their study samples and of the studies that did samples were predominately White, except for one study among African American breast cancer survivors [26]. African Americans are especially vulnerable to poor health outcomes and are disproportionately affected by many health conditions associated with poor diet quality, excess body weight, and physical inactivity [49]. More studies should specifically target racial and ethnic minorities, especially African Americans, to provide much-needed interventions and eliminate related cancer disparities.

## Conclusion

Web-delivered lifestyle interventions provide a means for delivering health behavior change programs to meet the growing needs of cancer survivors. Overall, the literature suggests that web-delivered lifestyle interventions have the potential to promote healthy lifestyle behavior among cancer survivors, especially increasing physical activity. However, future lifestyle interventions should address methodological issues outlined in this article (e.g., multiple behavior change, targeting minority and male survivors, post-intervention follow-up periods, and larger sample sizes).

## Figures and Tables

**Table 1:** Studies included in narrative review of web-based lifestyle interventions for cancer survivors.

First author, year [ref] Country	Baseline Sample Characteristics	Delivery Format	Intervention Description	Behavior(s) Targeted	Intervention Duration/ Follow-up	Theoretical Framework	Measure(s)	Major Findings
Rabin, 2011 [25] USA	Sedentary young adult cancer survivors N=18/mean age=32 years/56 % female/94 % White	Website	2-arm RCT Access to a physical activity website for cancer survivors, Step in Motion. Set PA goals and log Control was given info about three cancer survivor websites that did not provide info about PA.	PA	12 weeks	Transtheoretical Model Social Cognitive Theory	PA: PAR	Increased PA Baseline: 58.75 mins/week (44.54) vs. 39.00 (35.65), p=0.078 Follow-up: 161.25 (221.79) vs. 55.50 (77.48), p=0.48 Attrition Intervention: 5.6% at follow-up Control: 0% at follow-up
Hatchett,2013 [21] USA	Sedentary breast cancer survivors N=85/mean age= not reported/ aged 18+/100 % female/95 % White	Email	2-arm RCT Eight e-mails over 12 weeks Control group offered intervention after end of the trial	PA	12 weeks with measurements at 6 and 12 weeks	Social Cognitive Theory	PA: PAR	Increased PA Baseline: 0 days vs. 0 days Follow-up: 3.47 days (2.19) vs. 1.42 days (1.67), p<0.001 Attrition Overall: 12.9% at follow-up Intervention: 11.6% at follow-up Control: 14.3% at follow-up
Valle, 2013 [20] USA	Young adult cancer survivors N=86/mean age=32 years/91 % female/91 % White	Website and Facebook	2-arm RCT: FITNET or SC (self-help comparison) FITNET: Facebook group and study website access. Provides tools for goal setting, PA feedback, reminders to log PA SC: Assigned different Facebook group that provides information and weekly private messages with basic PA information.	PA	12 weeks	Social Cognitive Theory	PA: GLTEQ	Increased PA Fit NET: 187.6 mins/week (171.1) vs. 397.7 (778.4), p=0.009 Control: 199.3 mins/week (151.5) vs. 265.9 (228.1),p= 0.045 Attrition Overall: 18.6% at follow-up FITNET: 22.2% at follow up SC: 14.6% at follow-up
Bantum, 2014 [34] USA	Breast, colorectal, endometrium/uterine/ ovarian, non-Hodgkin’s lymphoma, lung, thyroid, and oral cancer survivors N= 352/ mean age = 51 years/ 82.1% female/ 87.2% White	Website	Two-arm delayed-treatment RCT Online workshop (website) to encourage healthy lifestyle behaviors	PA Diet Depression Fatigue	6 weeks with 6-month follow-up	N/A	PA: GLTEQ Diet: Block Food Frequency Questionnaire	Increased PA Intervention: 106 mins/week vs. 137 Control: 86.0 mins/week vs. 96.2 No change in diet Attrition Overall: 13.9% at 6-month follow-up Intervention: 16.5% at 6-month follow-up Control: 11.4% at 6 month follow-up
Berg, 2014 [31] USA	Young adults’ survivors of childhood cancer N= 24/mean age=23/ 71 % female/ 83% White	Website and email	One-arm, pre/post-test design Website & 12 modules delivered via email bi-weekly Tailored feedback	PA Reducing alcohol/ cigarette use	6 weeks	Theory of Reasoned Action	study questionnaire	No significant health behavior changes Attrition 4.2% at week 6; 20.8% at follow up
Frensham, 2018 [23] Australia	Sedentary adult cancer survivors N=91 mean age=66 years/52% female/ 96% White	Website	2-arm quasi-RCT 2 groups: Intervention and wait-list control Intervention: STRIDE website and weekly step goals. Website provides info on healthy lifestyles for cancer survivors	PA Secondary: Weight management	12 weeks with 3-month follow-up	Social Cognitive Theory	PA: New-Lifestyles Pedometer	Increased PA after intervention (week 12) with no changes after follow-up Baseline-STRIDE vs control: 7055 steps/day (2633) vs 6667 steps/day (2993); p=.56 Week 12:STRIDE vs control: 9274 steps/day (3579) vs 7499 steps/day (3320) p= .04 Follow-up: STRIDE vs control: 8437 steps/day (3487) vs 7333 steps/day (4044) p=.55 Attrition Overall: 18.6% at 12 weeks; 18.6% at 3 month follow up Intervention: 23.5% at 12 weeks; 23.5% at 3 months Control: 13.7% at 12 weeks; 13.7% at 3 months
Lee, 2014 [28] South Korea	Breast cancer survivors N=59/ mean age = 42 years/ 100% female	Website	Two-arm RCT: WSEDI vs control WSEDI: website containing tailored information on goal progress, action planning, goal setting and automatic feedback Control: 50-page educational booklet on diet and exercise	PA Diet	12 weeks	Transtheoretical Model	PA: 7-day diaries, study generated Diet: 3-day diet recall, diet quality index (DQI)	Increased PA Baseline--WSEDI vs control: 10 (33.3) vs. 10 (34.5) Follow-up--WSEDI vs control: 19 (65.5) vs. 10 (35.7), p<0.0001 Increased vegetable & fruit intake Improved diet quality Attrition Overall: 3.4% at follow-up WSEDI: 3.4% at follow-up Control: 3.3% at follow-up
Forbes, 2015 [22] Canada	Breast, colorectal, and prostate cancer survivors N=95/mean age=65.1 years/56 % female/99 % White	Website (online workshop) and emails	2-arm RCT UCAN: 9 module behavior change program using the UWALK website to track their PA and weekly emails Control group received usual care (no intervention).	PA	9 weeks	Theory of Planned Behavior	PA: GLTEQ	Increased PA Baseline-UCAN vs. control: 231 (269) vs. 212 (216) Follow-up-UCAN vs. control: 294 (354) vs. 241 (197) Attrition Overall: 11.6% at 9 weeks follow-up UCAN: 14.6% at 9 weeks follow-up control: 8.5% at 9 weeks follow-up
Hong, 2015 [27] USA	Older cancer survivors N=30/ median age = 69/ 70% female/73% White	Mobile application	One-arm, pre/post-test design iCanFit: Mobile-enabled web application with goal setting, activity tracking, tips, and social networking	PA	8 – 12 weeks	Theory of goal setting	PA: study questionnaire	Increased PA Not engaged in physical activity and have no plan: 3 participants (12%) vs 0, p=0.083 Not engaged in physical activity but plan to do so in 3 months: 5 participants (19%) vs 0, p=0.022 Engaged in physical activity occasionally, but not on a regular basis: 7 participants (27%) vs 5, p=0.77 Engaged in regular physical activity, but started less than 3 months: 0 participants vs 5 (19%), p=0.022 Engaged in regular physical activity and has been doing so for 3 months: 11 participants (42%) vs 15 (58%), p=0.043 Attrition 13.3% at follow up
McCarroll, 2015 [39] USA	Endometrial and breast cancer survivors N=50 mean age= 58/ 100% female/ 88% White	Website and mobile application	One-arm, pre/post-test design LoseIt! App: Commercially available food log, exercise log, daily body weight	Secondary: PA Diet Weight mgmt.	4 weeks	Social Cognitive Theory	PA: LoseIt app Diet: LoseIt app	PA increased initially, then decreased at 4 weeks-Week 1 and 2 to week 4 showed a trend towards a significance (p = 0.09) Baseline: 77.5185 (± 156.6) kcals expended and 22.7 (± 44.0) Week 1: 971.8 kcals (± 1105.4) and time 182.3 min (± 196.6), p = 0.001 Week 2: 973.0 kcals (± 953.7) and 200.2 min (± 216.1), p = 1.00 Week 3: 826.2 kcals (± 958.6) and 181.2 min (± 244.0), p = 1.00 Week 4: 632.0 kcals (± 909.8) and 127.0 min (± 185.3), p = 1.00 Weight loss 105.0 ± 21.8 kg versus 98.6 ± 22.5 kg, p = 0.000 BMI: 34.9 ± 8.7 kg/m^2^ versus 33.9 ± 8.4 kg/m^2^, p = 0.000 waist circumference: 108.1 ± 14.9 cm versus 103.7 ± 15.1 cm, p = 0.0006 No change in diet Attrition 30% at follow-up
Kanera, 2016 [35] Netherlands	Breast cancer survivors and others N=462/mean age= 55.9 years/ 79.9% female	Online workshop (website)	Two-arm RCT---KNW intervention and usual care KNW: self-management program	PA Diet Smoking	12 months	Theory of Planned Behavior Self-regulation theory I-Change Model	PA: SQUASH Diet: Adapted Dutch Standard Questionnaire on Food Consumption	Increased moderate PA---significant differences between arms: B = 117.738, p = .037, p fdr = .148, d = –0.25, f^2^ = .007 Short-term increase in vegetable intake Complete cases: B = 9.15, p = .027, p fdr = .148, d = −0.37, f^2^ = −.013; Intention-to-treat: B = 9.57, p = .023, p fdr = .160 Attrition Overall: 11.5% at 6 months : 17.5% at 12 months follow-up KNW: 18.6% at 6 months; 27% at 12 months Control: 4.3% at 6 months; 8.2% at 12 months
Kuijpers 2016 [33] Netherlands	Breast cancer survivors N=92/mean age = 49.5/ 100% female	Website	One-arm, pre/post-test design MijnAVL: website with access to medical records, personalized healthy lifestyle materials, PA feedback and support	PA	16 weeks	Social Cognitive Theory	PA: IPAQ	No significant change in PA Baseline: 2793 (MET-min/ week) Follow-up: 3724.2 (MET-min/ week) Attrition 0% at follow-up
Lynch, 2016 [30] USA	Overweight testicular and breast cancer survivors N=46/mean age=39/65% female/98% White	Website and email	One-arm, pre/post-test design Intervention: Commercially available website (Lean Eating)3 daily components: exercise, nutritional/behavioral modification, health lessons. Daily email reminders to log, coach assistant	Weight management Secondary: PA Diet	12 months with measurements at 0, 6, and 12 months	Social Cognitive Theory	Study generated questionnaire	No change in PA Body fat %: Baseline: 26.6% (4.7) 6 months: 24.4% (5.3), p=0.0004 12 months: 22.2% (4.9), p=0.002 Attrition 41% at 6 months; 51% at month 12
Puszkiewicz, 2016 [32] United Kingdom	Breast, prostate, and colorectal cancer survivors N=11/mean age = 45/ 82% female/ 82% White British	Mobile application	One-arm, pre/post-test design GAINFitness: Commercially available PA mobile application; goal setting, PA plans	PA Secondary: Weight mgmt.	6 weeks	N/A	PA: GLTEQ	Increased PA significant increase in strenuous PA between baseline (median=40, IQR=105) and follow-up (median=120, IQR=150), (z=−2.80, P=.002) Mild PA: baseline (median=150, IQR=90) and follow-up (median=80, IQR=120), (z=−2.21, P=.031 No change in BMI: 23.9 (5.2) vs 23.4 (5.0), p=0.828 Attrition 0% at follow-up
Golsteijn, 2018 [36] Netherlands	Colorectal or prostate cancer survivors N= 510/ mean age = 66 years/ 13% female	Website	2-arm RCT: OncoActive vs usual care Computer-tailored PA intervention with interactive website and print materials Provides feedback, goal setting	PA	16 weeks 2 month follow-up post intervention	I-Change Model Social Cognitive Theory Transtheoretical Model Health Belief Model Precaution Adoption Process Model Goal setting theories Health action process approach Theories of self-regulation	PA: ActiGraph Accelerometer, SQUASH	Increased PA Acti Graph Baseline-Onco Active vs usual care: 271 (211) vs. 293 (230), p=0.30 Follow-up—Onco Active vs usual care: 331 (234) vs. 301 (219), p-0.006 SQUASH Baseline—Onco Active vs usual care: 780 (721) vs. 873 (764), p=0.29 Follow-up—Onco Active vs usual care: 1145 (883) vs. 213 (943), p<0.001 Attrition Overall: 4.4% at 16 weeks; 7.3% at 2 month follow up; Intervention: 6.0% at 16 weeks 9.6% at 2 month follow-up post intervention Control: 2.6% at 16 weeks 4.8% at 2 month follow-up
Paxton, 2017 [26] USA	Breast cancer survivors N=71/mean age = 52 years/ 100% female/ 83% African American	Website & Email	Two-arm RCT Parallel-group feasibility study ALIVE: Randomized to physical activity or diet email-based intervention	PA Diet	12 weeks	Social Cognitive Theory TTM Goal-setting theory Social marketing	PA: study generated questionnaire Diet: study generated questionnaire	Increased PA PA arm: +165 mins/week (68) vs Diet arm: +75 mins/week (62), p<0.001 Diet---saturated fat PA arm: −1.0 grams/day (1.3) vs Diet arm: −0.8 (1.2), p=0.46 Diet---vegetables and fruits PA arm: +0.6 cups/day (0.3)vs Diet arm: +1.0 (0.3), p=0.35 Attrition Overall: 38% at 3 months PA arm: 41.2% at 12 weeks Diet arm: 35.1% at 12 weeks
Short, 2017 [37] Australia	Breast cancer survivors N = 492/ mean age = 55 years / 100% female	Website	3-arm RCT Three-arms: (1) a computer-tailored three-module intervention delivered monthly; (2) a computer-tailored three-module intervention delivered weekly (over the first 3 weeks) or (3) a computer-tailored single module intervention	PA	12 weeks	Social Cognitive Theory	PA: GLTEQ	Increased PA in each arm from baseline to follow-up: Arm 1: 90.08 mins/week (106.63) vs 216.99 (219.99), p < 0.05 Arm 2: 97.17 mins/week (124.10) vs 186.08 (157.89), p < 0.05 Arm 3: 96.15 mins/week (119.63) vs 186.05 (172.56), p < 0.05 Attrition Overall: 68 % at 12 weeks follow-up Arm 1: 71 % Arm 2: 73 % Arm 3: 61 %
Uhm, 2017 [38] South Korea	Breast cancer survivors N=356/mean age = 50.3/ 100% female	Mobile application	Quasi two-arm RCT Smart After Care: Access to mobile PA application and pedometer Control: Treatment as usual-given PA brochure	PA QoL Secondary: BMI (weight management	12 weeks	N/A	PA: IPAQ-SF	Increased PA (METs) Intervention---Baseline 2050.6 ± 2182.2 vs Follow-up 3026.9 ± 2489.5, p < 0.05 Control---Baseline 2091.5 ± 1811.2 vs Follow-up 2560.4 ± 2354.9, p < 0.05 No change in BMI: Intervention--Baseline 23.3 ± 3.1 vs Follow-up 23.3 ± 3.1 Control---Baseline 23.3 ± 3.3 vs Follow-up 23.3. ± 3.4 Attrition Overall: 3.9% at 6-weeks 4.8% at 12-weeks Intervention: 5.6% at 6 weeks 6.7% at 12 weeks Control: 2.3% at 6 weeks 2.8% at 12 weeks
Trinh, 2018 [24] Canada	Prostate cancer survivors N=46/mean age=73.2 years/100% male/ 80.4% White	Mobile application	One-arm, pre-/post-test design Feasibility study Rise Tx: Access to application aimed at increasing PA	Secondary: PA	12 weeks 3-month follow-up	N/A	PA: Acti Graph Accelerometer	Increased PA significantly from baseline to 12 weeks post Baseline: 93.1 mins/week (14.5) 12 weeks: 137.1 mins/week (22.8), p = .010 3-months post-intervention: 122.1 (23.9) mins/week, p = .18 Attrition 4.7% at follow-up

**Abbreviations:** BMI: Body Mass Index; GLTEQ: Godin Leisure-Time Exercise Questionnaire; IPAQ: International Physical Activity Questionnaires; IPAQ-SF: International Physical Activity Questionnaires short form PA: Physical activity; METs: Metabolic equivalents; N/A: Not applicable; PAR: Seven-Day Physical Activity Recall; QoL: Quality of Life; RCT: Randomized control trial; SQUASH: Short Questionnaire to Assess Health-Enhancing

* Weight was measured using a scale.

## Abbreviations:

PRISMA: Preferred Reporting Items for Systematic Reviews and Meta-Analyses
DQI: Diet Quality Index
SQUASH: Short Questionnaire to Assess Health-Enhancing
PAR: Physical Activity Recall
SQUASH: Short Questionnaire to Assess Health-Enhancing
IPAQ: International Physical Activity Questionnaires
CAPS: Cross-Cultural Activity Participation Study
METs: Metabolic Equivalents
SCT: Social Cognitive Theory
TTM: Transtheoretical Model
TPB: Theory of Planned Behavior
HBM: Health Belief Model
PAPM: Precaution Adoption Process Model
